# IL-10 and TGF-β, but Not IL-17A or IFN-γ, Potentiate the IL-15-Induced Proliferation of Human T Cells: Association with a Decrease in the Expression of β2m-Free HLA Class I Molecules Induced by IL-15

**DOI:** 10.3390/ijms25179376

**Published:** 2024-08-29

**Authors:** Leila H. Duarte, Hugo A. Peixoto, Elsa M. Cardoso, André J. Esgalhado, Fernando A. Arosa

**Affiliations:** 1 CICS-UBI, Health Sciences Research Centre, University of Beira Interior, 6200-506 Covilhã, Portugal; leiladuartebbe@hotmail.com (L.H.D.); hugopeixoto97@hotmail.com (H.A.P.); elsa.cardoso@ipg.pt (E.M.C.); andre.esgalhado@fcsaude.ubi.pt (A.J.E.); 2 ESS-IPG, School of Health Sciences, Polytechnic of Guarda, 6300-559 Guarda, Portugal; 3 FCS-UBI, Faculty of Health Sciences, University of Beira Interior, 6200-506 Covilhã, Portugal

**Keywords:** cytokines, CD8+ T cells, proliferation, HLA class I molecules, open conformers, W6/32, HC-10

## Abstract

IL-15 is a homeostatic cytokine for human T and NK cells. However, whether other cytokines influence the effect of IL-15 is not known. We studied the impact that IL-10, TGF-β, IL-17A, and IFN-γ have on the IL-15-induced proliferation of human T cells and the expression of HLA class I (HLA-I) molecules. Peripheral blood lymphocytes (PBLs) were labeled with CFSE and stimulated for 12 days with IL-15 in the absence or presence of the other cytokines. The proportion of proliferating T cells and the expression of cell surface HLA-I molecules were analyzed using flow cytometry. The IL-15-induced proliferation of T cells was paralleled by an increase in the expression of HC-10-reactive HLA-I molecules, namely on T cells that underwent ≥5–6 cycles of cell division. It is noteworthy that the IL-15-induced proliferation of T cells was potentiated by IL-10 and TGF-β but not by IL-17 or IFN-γ and was associated with a decrease in the expression of HC-10-reactive molecules. The cytokines IL-10 and TGF-β potentiate the proliferative capacity that IL-15 has on human T cells in vitro, an effect that is associated with a reduction in the amount of HC-10 reactive HLA class I molecules induced by IL-15.

## 1. Introduction

Cytokines are small proteins secreted by cells of both the innate and adaptive immune systems that regulate a diverse array of biological processes within and outside the immunological system. IL-10 is an immune-related cytokine capable of inhibiting co-stimulatory signals via CD28 in naïve T cells, reducing the differentiation of Th1 cells and the development of dendritic cells, suppressing the activity of Th17 cells, and preserving Foxp3 expression in Tregs [1,2,3,4,5,6,7]. In addition, IL-10 has also been described as having inhibitory and stimulatory effects on human CD8+ T cells. While the inhibition of CD8+ T cells is an indirect effect resulting from the down-modulation of costimulatory molecules and MHC-I molecules by antigen-presenting cells, stimulation is associated with the expression of the IL-2 receptor α chain [8,9,10]. TGF-β is a known regulator of a variety of processes, including cell activation, proliferation, and differentiation. Thus, TGF-β has been shown to inhibit T-cell differentiation, promote the formation and expansion of Treg cells, impair the maturation of dendritic cells, promote *trans*-differentiation between Th17 and Tregs and vice versa, and inhibit the cytolytic activity of cytotoxic T lymphocytes [11,12,13,14,15,16,17,18]. In contrast to IL-10 and TGF-β, IFN-γ and IL-17A are immune-related cytokines capable of causing cell activation and inflammation. IFN-γ is primarily produced by NK, NKT, Th1, and cytotoxic CD8+ T cells [19]. In addition to its well-known role in augmenting MHC expression on a variety of immune and non-immune cells, IFN-γ has been long known as a cytokine with a marked direct effect on the activation of human CD8+ T cells [20,21]. Although IL-17A shares some biological effects with IFN-γ, the scientific data of its effects on T cells are scarce or lacking. Thus, in addition to its involvement in the activation of a variety of non-immune cells, IL-17 alone or in combination with IFN-γ recruits and activates innate cells at sites of inflammation, therefore contributing to augmenting the inflammatory process [22,23].

Despite this body of knowledge, the effect that these cytokines have on the antigen-independent activation of CD3+ T cells, namely by cytokines such as IL-15, is presently unknown. We have previously shown that IL-15 functions as a homeostatic cytokine that regulates the activation and expansion of naïve CD8+ T cells in vitro [24,25,26]. We, and others, have also shown that T-cell activation leads to changes in the physiological equilibrium between closed (i.e., β2m-associated, W6/32-reactive) and open (i.e., β2m-free, HC-10-reactive) HLA-I conformers [27,28]. The existence of open HLA-I conformers is physiologically and functionally relevant in normal and clinical settings, such as during normal T-cell activation [27], transplantation [29], autoimmunity [30], and malignancy [31,32]. This is due to the special molecular features of these molecules, which allow them to interact in *cis* and *trans* with NK receptors and growth factor receptors (reviewed in [33]). However, whether IL-15 itself or in combination with any of the aforementioned immune-related cytokines has an effect on the expression levels of open HLA-I conformers on the cell surface of activated T cells is not known. Since IL-15 and the aforementioned cytokines are likely to share the same microenvironments at different phases of the inflammatory process, we are interested in examining the effects of IL-10, TGF-β, IFN-γ, and IL-17A on in vitro IL-15-activated human T cells, namely on their proliferative capacity and on the physiological equilibrium between closed and open HLA-I conformers.

## 2. Results

**Figure 1:**
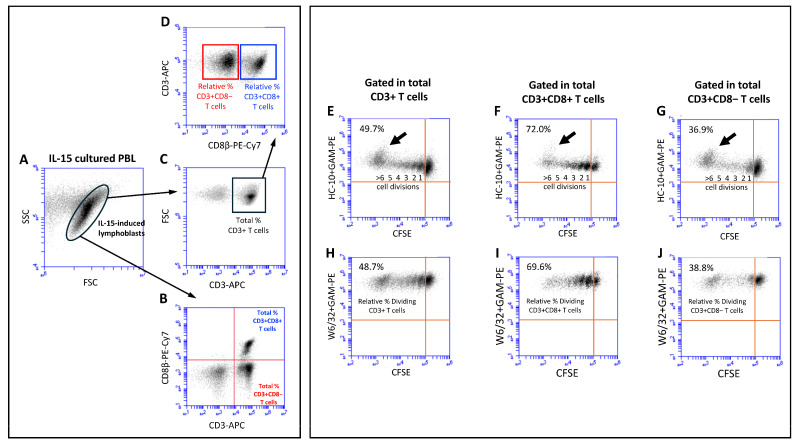
**Gating strategies to study the phenotype and expression of the HC-10 and W6/32 epitopes in lymphoblasts induced by IL-15**. (**Left panel**): Fresh PBLs were isolated, labeled with CFSE, and cultured for 12 days in the presence of IL-15, which was added at days 0 and 6, as indicated in Section 4. (**A**) Dot plot shows FSC versus SSC parameters of lymphoblasts at day 12 of culture with IL-15, illustrating the blast transformation of PBLs in response to IL-15. Small and larger blast cells can be observed. (**B**) Dot plot shows CD3 versus CD8 expression on gated IL-15-induced lymphoblasts, allowing for the determination of the percentage of total CD3+CD8+ T cells (upper right quadrant, URQ) and CD3+CD8− T cells (lower right quadrant, ULQ). (**C**) Dot plot shows CD3 versus FSC on gated IL-15-induced lymphoblasts, allowing for the determination of the percentage of total CD3+ T cells. (**D**) Dot plot shows CD8 versus CD3 expression on gated total CD3+ T cells (black square), allowing for the determination of the relative percentages of CD3+CD8+ T cells (blue square) and CD3+CD8− T cells (red square). (**Right panel**): Upper dot plots show CFSE halving versus HC-10 expression in total CD3+ T cells (**E**), total CD3+CD8+ T cells (**F**), and total CD3+CD8− T cells (**G**). The percentage of dividing cells (ULQ) and the number of cycles of cell divisions are indicated. Black arrow highlights the most dividing cells (≥5–6 division cycles). Lower dot plots show CFSE halving versus W6/32 expression in total CD3+ T cells (**H**), total CD3+CD8+ T cells (**I**), and total CD3+CD8− T cells (**J**). The percentage of dividing cells (ULQ) are indicated. These dot plots allowed us to determine the relative percentage of dividing CD3+ T cells, CD3+CD8+ T cells, and CD3+CD8− T cells.

**Figure 2:**
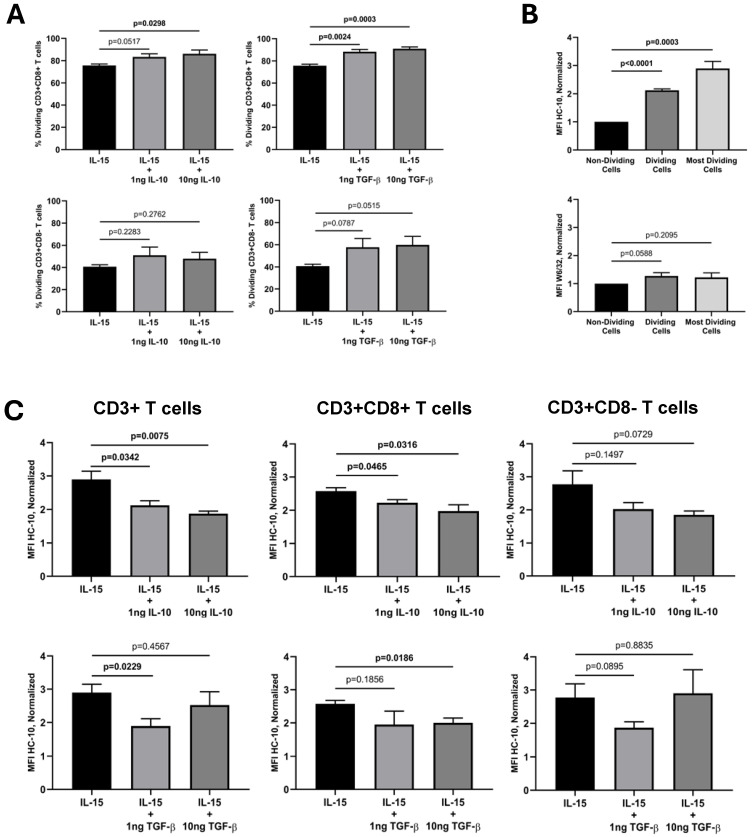
**Effects of IL-15, IL-10, and TGF-β on the percentage of dividing T cells and the expression of HC-10 and W6/32 epitopes**. Fresh PBLs were isolated and cultured, as indicated in the legend of Figure 1, in the absence or presence of IL-10 and TGF-β. Twelve-day activated PBLs were labeled with W6/32 + GAM-PE or HC-10 + GAM-PE, followed by anti-CD3 and anti-CD8β antibodies, as indicated in the Material and Methods, and analyzed using flow cytometry. (**A**) Upper graphs show the relative percentage of dividing CD3+CD8+ T cells (Mean ± SEM, n = 4) on gated lymphoblasts upon culture with IL-15 alone or in the presence of 1 ng and 10 ng of IL-10 and TGF-β. Lower graphs show the relative percentage of dividing CD3+CD8− T cells (Mean ± SEM, n = 4) on gated lymphoblasts upon culture with IL-15 alone or in the presence of 1 ng and 10 ng of IL-10 and of TGF-β. (**B**) The graphs show the normalized HC-10 (upper graph) and W6/32 MFI (lower graph) values (Mean ± SEM, n = 4; see Section 4) on non-dividing CD3+ T cells, total dividing CD3+ T cells and the most dividing CD3+ T cells (≥5–6 cycles of cell division). (**C**) Upper graphs show the normalized HC-10 MFI values (Mean ± SEM, n = 4) on the most dividing CD3+, CD3+CD8+, and CD3+CD8− T cells upon culture with IL-15 alone or in the presence of 1ng and 10ng of IL-10. Lower graphs show the normalized HC-10 MFI values (Mean ± SEM, n = 4) on the most dividing CD3+, CD3+CD8+, and CD3+CD8− T cells upon culture with IL-15 alone or in the presence of 1 ng and 10 ng of TGF-β. All amounts of cytokines indicated are in ng/mL. *p* values are indicated.

To elucidate the impact of immune-related cytokines on the IL-15 activation and proliferation of ex vivo human T cells, fresh peripheral blood lymphocyte (PBL) preparations were cultured in vitro for 12 days in the presence of IL-15 alone (10 ng/mL) or in combination with IL-10, TGF-β, IL-17A, and IFN-γ at two different concentrations (1 ng/mL and 10 ng/mL). While IL-15 was added at the start of the culture and six days later, the other cytokines were only added at the start of the culture. Figure 1 (left panel) shows the gating strategy to determine the total and relative percentages of CD3+, CD3+CD8+, and CD3+CD8− T cells in cultures of PBL in the presence of IL-15 for 12 days. IL-15 activation of PBL induced the formation of a population of lymphoblasts (Figure 1A) that contained a mixture of CD3+CD8+ T cells, CD3+CD8− T cells, and lymphocytes negative for both T-cell markers (Figure 1B), which allowed us to calculate the percentage of the two T-cell populations. Cells within the IL-15-induced lymphoblasts were predominantly CD3+ T cells (Figure 1C, usually >75%). After gating in the CD3+ T cells (black square), we could determine the relative percentages of CD3+CD8+ T cells (blue square) and CD3+CD8− T cells (red square) at the end of the culture (see Figure 1D). As previously shown by us [25,26], the percentage of CD3+CD8+ T cells that divided in response to IL-15 outnumbered the percentage of CD3+CD8− T cells in a ratio of approximately 2:1 (75.7% vs. 40.8%, *p* = 0.0001; n = 4) (Figure 1, right panel, compare dot-plots F/I with dot-plots G/J). The superior proliferation of CD3+CD8+ T cells was seen with all combinations of cytokines (Supplementary Figure S1A). The same type of analysis was performed in cultures with combinations of IL-15 and the other cytokines. Interestingly, the addition of 10 ng/mL of IL-10 and 1 ng/mL and 10 ng/mL of TGF-β to the IL-15-stimulated PBL cultures significantly increased the relative percentage of CD3+CD8+ T cells, but not of CD3+CD8− T cells, at the end of the culture (Figure 2A). In marked contrast, IL-17A and IFN-γ had no effect on the IL-15-induced CD3+CD8+ T cell proliferation (Supplementary Figure S1B).

Next, we were interested in examining the expression of HLA-I molecules at the cell surface of the activated and dividing CD3+ T cells and the subsets within. Previous studies have shown that mature β2m-free HLA-I molecules, also known as open HLA-I conformers, are expressed at the cell surface by antigen-activated normal T cells and transformed T- and B-cell lines and are involved in regulating key plasma membrane-associated events, including intracellular pathways mediated by signaling receptors and receptor endocytosis [27,31,34,35,36]. Thus, we wanted to ascertain whether IL-15 alone, or its combination with the other cytokines, influenced the expression of HLA-I molecules, either as β2m-associated, W6/32-reactive (i.e., closed) HLA-I conformers or as β2m-free, HC-10-reactive (i.e., open) HLA-I conformers. Figure 1 (right panel) shows the gating strategy to determine the absolute mean fluorescence intensity (MFI) expression values of HC-10-reactive and W6/32-reactive HLA-I forms in CD3+ T cells (Figure 1E and Figure 1H, respectively), CD3+CD8+ T cells (Figure 1F and Figure 1I, respectively), and CD3+CD8− T cells (Figure 1G and Figure 1J, respectively) in the dividing (CFSE halving) cells. The analysis of the expressions of closed and open HLA-I conformers at the cell surface of the dividing CD3+ T cells revealed interesting results. While the expression of closed HLA-I conformers remained steady during the cell divisions (Figure 1H), the expression of open conformers consistently increased with each cycle of cell division, being maximal in CD3+ T cells that underwent ≥5–6 cell division cycles (see Figure 1E; see black arrow). These results were observed both in CD3+CD8+ and CD3+CD8− T cells (Figure 1F,G versus Figure 1I,J).

A comparison of the absolute MFI values for W6/32 and HC-10 in the different culture conditions between the non-dividing cells (NDCs), the total dividing cells (DCs), and the pool of the most dividing cells (MDCs) revealed a high variability in the MFI values of HC-10-reactive, but not W6/32-reactive HLA-I forms, i.e., the open conformers, between experiments (Table 1). Indeed, the coefficient of variation (CV) for the absolute HC-10 MFI values in the MDCs was approximately three-fold higher (range, 46.5–79.3) than the absolute W6/32 MFI values (range, 8.4–28.7). Nevertheless, a steady increase in the expression of HC-10-reactive forms between the NDCs, DCs, and MDCs was consistently observed in every experiment (Table 1 and Supplementary Figure S2A). It is noteworthy that while the absolute W6/32 MFI values in non-dividing cells in the different culture conditions ranged between 240,816 and 830,854 auf, the absolute HC-10 MFI values in non-dividing cells ranged between 2324 and 16,399 auf. On average, the level of expression of closed HLA-I conformers was about 35-fold higher than the level of expression of open HLA-I conformers, with the background fluorescence levels being below 1000 auf (Supplementary Figure S2B). Given the differences in the absolute MFI values obtained between experiments and in order to overcome the challenge we normalized the absolute MFI values in the dividing CD3+ T cells in relation to the non-dividing cells, i.e., the fraction of CD3+ T cells that did not show CFSE halving, as indicated in Section 4. We have conducted this analysis in previous studies for other cell surface markers [37].

The normalized HC-10 (open HLA-I conformer) MFI values in IL-15-activated CD3+ T cells were statistically significantly higher (approx. two-fold increase; *p* < 0001, n = 4) when the total fraction of dividing CD3+ T cells was compared to the non-dividing cells. This increase was even more striking (approx. three-fold increase; *p* = 0.003, n = 4) when only the pool of the most dividing CD3+ T cells was compared (Figure 2B, upper graph). This pattern mirrored the one with the absolute MFI values (see Supplementary Figure S2A). Importantly, the increase in open HLA-I conformers was observed both in CD3+CD8+ and CD3+CD8− T cells (Supplementary Figure S2C). Regarding the normalized W6/32 (closed HLA-I conformer) MFI values in IL-15-activated CD3+ T cells, no significant changes were observed (Figure 2B, lower graph). However, IL-15 induced a slight but significant increase in the expression of closed HLA-I conformers by CD3+CD8− T cells (Supplementary Figure S2C). Interestingly, when the same analysis was conducted for the combination of IL-15 with the other cytokines, IL-10 and TGF-β significantly reverted the marked increase in open HLA-I conformers induced by IL-15 in the most dividing cells. This reversion was statistically significant in CD3+ T cells (IL-10 at 1 ng/mL and 10 ng/mL; TGF-β at 1 ng/mL) and CD3+CD8+ T cells (IL-10 at 1 ng/mL and 10 ng/mL; TGF-β at 10 ng), but not in CD3+CD8− T cells (Figure 2C). Indeed, this effect appeared to be reduced when using a high concentration of TGF-β (10 ng/mL) for CD3+ and CD3+CD8− T cells. Likewise, IL-17A and IFN-γ did not have a significant effect on the expression of HC-10-reactive molecules induced by IL-15 (Supplementary Figure S2D).

## 3. Discussion

This study aimed to elucidate the impact of four immune-related cytokines, namely IL-10, TGF-β, IL-17A, and IFN-γ, on the IL-15-induced activation of human CD3+ T cells. Some of these cytokines play important roles in the biology of T cells, both in vitro and in vivo. Moreover, some of them are presently being used as part of immunotherapies aimed at expanding/inhibiting cytotoxic/autoreactive T cells to treat cancer, autoimmune disorders, and downplaying inflammation in chronic disorders [38,39,40,41,42]. Thus, ascertaining whether these cytokines have an enhancing or inhibitory effect on the IL-15-mediated activation and proliferation of CD3+ T cells and subsets within may provide important insights in our understanding of the biology of these cytokines. IL-15 is known to be involved in the activation and proliferation of human CD8+ T cells, with some of them differentiating into effector memory CD8+ T cells expressing variable levels of CD45RA (i.e., CD8+ T_EMRA_) akin to a variety of NK receptors and expressing CD8⍺⍺ homodimers [25,26,37]. However, this is the first time that the IL-15-mediated activation of CD3+ T cells also induced the expression of open HLA-I conformers at the plasma membrane, adding to the body of knowledge indicating that these HLA-I conformers are expressed as a result of cell activation, proliferation and differentiation regardless of the nature of the stimuli [37]. In this regard, it is important to note that the expression of open HLA-I conformers is highly variable and is influenced by the activation and metabolic status of the cells. Thus, while resting cells barely express open HLA-I conformers, activated cells and malignant cell lines express highly variable levels [27,31]. Even ex vivo lymphocytes and monocytes vary in the expression of HC-10-reactive forms when healthy controls and patients with malignancies are studied [32], with possible physiological implications, from the modulation of intracellular signals to the regulation of transferrin receptor endocytosis [27,31,33,34,35].

Of note, the potentiating effect that the presence of IL-10 and TGF-β had on the proliferation of the IL-15-activated CD3+CD8+ T cells was associated with a statistically significant reduction in the expression of the HLA-I conformers not associated with β2m. These results are concordant with the lack of effect observed with IL-17A and IFN-γ both in potentiating IL-15-mediated T-cell proliferation and in increasing HC-10-reactive open HLA-I conformers. A lower expression of open conformers in IL-15 cultures with IL-10 and TGF-β can also be interpretated as a result of a higher number of *cis*-associations with other cell surface receptors that are important for regulating T-cell proliferation. Earlier studies demonstrated the existence of these *cis*-associations by adding β2m extracellularly, which restored normal closed conformer expression ([33] and reference herein). Since IL-10 and TGF-β are cytokines associated with immunosuppressive processes, their effects on the proliferation of CD3+CD8+ T cells and on the increase in the percentage of CD3+CD8+ T cells at the end of the culture are apparently contradictory. TGF-β is best known for influencing T-cell differentiation in combination with other cytokines [39,42] but not necessarily their proliferation. IL-10, on the other hand, is known for inhibiting or stimulating T-cell proliferation depending on the cell status at the time of exposure and whether an APC is present or not [8,9]. It is possible that in our culture conditions, IL-15 may induce, at some point, an increase in the expressions of IL-10 and TGF-β receptors at the plasma membrane of CD3+CD8+ T cells, allowing for a more pronounced effect of these cytokines. The difference observed between CD3+CD8+ T cells and CD3+CD8− T cells when using a high concentration of TGF-β (10 ng/mL) could be due to differences in the expression of the Krüppel-like factor KLF10, which appears to impact differently on TGF-β receptor II expression and function in CD4+ and CD8+ T cells [43], or because high TGF-β concentrations may exert a direct inhibitory effect on the expression open HLA-I conformers on CD3+CD8+ T cells but not on CD3+CD8− T cells, as reported for IFN-γ on antigen presenting cells [44]. Alternatively, these cytokines may enhance the generation of CD8+ T_EMRA_ cells and CD8αα T cells, which contain subsets of suppressor/regulatory T cells [37,45,46]. In either case, IL-10 and TGF-β emerge as modulators of the physiological equilibrium between closed (i.e., β2m-associated, W6/32-reactive) and open (i.e., β2m-free, HC-10-reactive) HLA class I conformers at the cell surface of activated CD3+ T cells, with likely functional implications [33]. Further studies on the underlying mechanisms responsible for the effect of IL-10 and TGF-β on the IL-15-induced activation and proliferation of human CD3+ T are warranted.

Despite the innovative nature of some of the results presented here, we acknowledge several limitations in the study, namely the low number of samples studied, together with the constraints regarding the phenotypic and functional characterizations of the IL-15-activated T cells. Certainly, studies addressing the physiological impact of the expression of high levels of open HLA-I conformers (HC-10-reactive) by the dividing and most dividing T cells are needed. Nevertheless, we think that the data presented in this manuscript could drive new studies addressing the full phenotypic and functional characterizations of these IL-15-activated CD3+ T cells.

## 4. Materials and Methods

### 4.1. Ethics Statement

Human peripheral blood mononuclear cells (PBMCs) were obtained from buffy coats of anonymous, healthy regular blood donors kindly provided by the Centro do Sangue e da Transplantação de Coimbra (CST-C, Portugal) under a protocol approved by the Portuguese Institute of Blood and Transplantation (IPST, IP, Lisbon), the University of Beira Interior (UBI), and the Faculty of Health Sciences (FCS-UBI). The study protocol was approved by the Ethics Committee of the University in accordance with the Declaration of Helsinki (Ref. Number CE-UBI-Pj-2017-012).

### 4.2. Cells Isolation, CFSE Labeling and Culture Conditions

PBMCs were isolated, as previously described by us, from buffy coats after centrifugation over Lymphoprep (STEMCELL Technologies, Saint-Egrève, France). Contaminating red blood cells were lysed in lysis solution (10 mM Tris and 155 mM NH_4_Cl, pH 7.4) for 10 min at 37 °C. Enriched peripheral blood lymphocytes were obtained after the incubation of PBMCs in Petri dishes for 1 h at 37 °C and 5% CO_2_ to deplete adherent cells, namely monocytes, as previously described [27]. The recovered non-adherent cell suspensions were routinely >80% CD3+ T cells and are referred to as PBLs. Freshly isolated PBLs were immediately labeled with the CellTrace^TM^ CFSE Cell Proliferation kit (Thermo-Fisher Scientific, Waltham, MA, USA) at a final concentration of 5 μM for 5 min at room temperature (RT) in phosphate-buffered saline (PBS) with occasional mixing, followed by three washes with RPMI-1640 medium (Thermo-Fisher Scientific) containing 10% heat-inactivated fetal bovine serum (FBS). Then, CFSE-labeled PBLs (1.0 × 10^6^/mL) were cultured in 24-well plates (Greiner Bio-One, Austria) in RPMI-1640 GlutaMAX medium (Thermo-Fisher Scientific) supplemented with 5% human serum (Sigma-Aldrich, Burlington, MA, USA) and 1% antibiotic–antimycotic solution (Sigma-Aldrich) at 37 °C, 5% CO_2_, and 95% humidity for 12 days. The PBLs were cultured in the presence of IL-15 alone (10 ng/mL) added at days 0 and 6, or in combination with 1 ng/mL and 10 ng/mL of IL-10, TGF-β, IFN-γ, and IL-17A (R&D Systems, Minneapolis, MN, USA), added at day 0.

### 4.3. Flow Cytometry Studies

For cell surface staining, approximately 0.5 × 10^6^ cells were incubated in 96-well round-bottom plates at 4 °C in the dark for 30 min with combinations of different unconjugated and fluorochrome-conjugated antibodies diluted in staining solution (PBS, 0.2% BSA, and 0.1% NaN_3_). Briefly, cells were first separately incubated with the unconjugated W6/32 antibodies (Thermo-Fisher Scientific), which recognize a monomorphic epitope on all classical HLA class I heavy chains, dependent on the presence of β2m ([28] and references herein), or HC-10 antibodies (Nordic-MUbio), which recognize HLA class I heavy chains not associated with β2m and peptides and having the peptide sequence PxxWDR in the α1 domain ([28] and references herein), followed by incubation with PE-conjugated goat anti-mouse antibodies (GAM-PE, BioLegend, San Diego, CA, USA). After washing, W6/32- and HC-10-labeled cells were incubated with APC-conjugated mouse anti-CD3 (CD3-APC, BioLegend) and PE-Cy7-conjugated mouse anti-CD8β (CD8β-PE-Cy7; Thermo Fisher Scientific) antibodies. Irrelevant IgG2a-PE-Cy7 mouse antibodies were used as the control for background fluorescence (Thermo-Fisher Scientific). After washing, a minimum of 20,000 events were acquired in a BD Accuri C6 (BD Biosciences, Franklin Lakes, NJ, USA). The labeled cells were analyzed using BD Accuri C6 software (BD Biosciences).

### 4.4. Quantification of Cell Proliferation

T-cell divisions were determined in all cells that sequentially decreased in the CFSE fluorescence intensity (CFSE halving) after the period of culture, as shown, for example, in Figure 1E–G (upper left quadrants). In all the experiments performed, CFSE halving allowed us to distinguish the different cycles of cell division. To quantitate changes in the percentages of CD3+, CD3+CD8+, and CD3+CD8− T cells and in the expression of W6/32+ and HC-10+ HLA-I conformers, electronic regions were created around CD3+, CD3+CD8+, and CD3+CD8− T cells, and the mean fluorescence intensity (MFI) values of the two HLA-I forms were determined. MFI values of cells that did not divide were used to normalize the MFI values of the dividing cells as follows: (MFI dividing cells/MFI non-dividing cells) × 100.

### 4.5. Statistical Analysis

For flow cytometry data, statistical analysis was performed using the software Graph Pad Prism 8 (GraphPad Software Inc., Boston, MA, USA). Continuous variables were expressed as the Mean ± Standard Error of the Mean (SEM). Differences between the means of two continuous variables were analyzed using Student’s *t*-test. All the data were checked for normality. The coefficient of variation (CV) within samples was calculated as follows: (standard deviation/mean) × 100. Statistical significance was defined as *p* < 0.05. Normalization of the HC-10 and W6/32 MFI values was performed by comparing the values obtained in the total dividing and most dividing T cells with the values in non-dividing T cells.

## Figures and Tables

**Table 1:** Effects of the cytokine combinations on the absolute MFI expression values for W6/32 and HC-10-reactive HLA-I conformers in the cell surface of the IL-15-activated CD3+ T cells ^#^.

Cytokines	Exp.	Absolute W6/32 MFI Values (auf)			Absolute HC-10 MFI Values (auf)		
		NDC	DC	MDC	NDC	DC	MDC
**IL-15**	1	582,286	670,306	645,073	2434	3867	7855
	2	279,225	597,143	473,002	4670	9595	10,396
	3	308,030	328,094	348,512	6624	14,539	19,520
	4	369,864	394,137	383,243	12,406	25,252	40,912
Mean ± SEM		384,851 ± 68,474	497,420 ± 81,226	462,458 ± 66,281	6534 ± 2136	13,313 ± 4538	19,671 ± 7510
CV		35.6	32.7	28.7	65.4	68.2	76.4
**+1 ng IL-10**	1	690,499	696,209	592,428	2563	4973	4730
	2	313,580	577,480	534,129	5380	10,577	10,729
	3	299,515	410,883	438,325	6835	12,862	15,825
	4	531,147	611,837	628,632	12,310	18,768	29,602
Mean ± SEM		458,685 ± 93,710	574,102 ± 59,851	548,379 ± 41,529	6772 ± 2048	11,795 ± 2855	15,222 ± 5303
CV		40.9	20.9	15.21	60.5	48.4	69.7
**+10 ng IL-10**	1	561,224	696,209	609,248	2324	3548	4292
	2	280,539	479,845	542,653	5251	9570	9474
	3	320,450	353,883	498,150	7532	10,370	16,433
	4	488,084	563,177	537,863	14,953	20,244	27,516
Mean ± SEM		412,574 ± 66,907	523,279 ± 71,925	546,979 ± 23,028	7515 ± 2699	10,933 ± 3457	14,429 ± 5022
CV		32.4	27.5	8.4	71.8	63.2	69.6
**+1 ng TGF-β**	1	506,734	350,418	615,149	4153	4094	6223
	2	285,272	481,154	525,100	7249	9929	12,392
	3	273,987	479,845	383,225	7657	9590	19,352
	4	305,044	432,657	445,050	11,863	16,577	22,266
Mean ± SEM		342,759 ± 55,034	436,019 ± 30,682	492,131 ± 50,247 *	7731 ± 1584	10,048 ± 2554	15,058 ± 3600 *
CV		32.1	14.1	20.4	41.0	50.8	47.8
**+10 ng TGF-β**	1	449,394	497,858	596,349	2870	3165	6614
	2	240,816	473,261	491,321	5692	9772	12,335
	3	260,754	364,314	436,399	6025	10,912	22,536
	4	275,224	490,359	490,673	9598	15,586	18,287
Mean ± SEM		306,547 ± 48,135	456,448 ± 31,140	503,686 ± 33,462 *	6046 ± 1379	9859 ± 2561	14,943 ± 3476 *
CV		31.4	13.6	13.3	45.6	52.0	46.5
**+1 ng IL-17A**	1	450,968	915,144	494,647	5152	7059	8190
	2	346,997	570,424	643,442	4946	7998	12,446
	3	336,243	433,139	425,421	6515	15,365	18,809
	4	441,588	416,545	486,243	16399	24,189	36,747
Mean ± SEM		393,949 ± 30,352	583,813 ± 115,701	512,438 ± 46,311	8253 ± 2738	13,653 ± 3973	19,048 ± 6290
CV		15.4	39.6	18.1	66.3	58.2	66.0
**+10 ng IL-17A**	1	830,854	934,290	863,447	3646	4992	8420
	2	344,445	590,201	630,329	4392	8418	11,873
	3	394,027	494,165	477,729	7869	15,631	13,492
	4	478,755	574,801	532,176	15043	25,087	40,330
Mean ± SEM		512,020 ± 109,835	648,364 ± 97,607	625,920 ± 85,238	7738 ± 2603	13,532 ± 4444	18,529 ± 7344
CV		42.9	30.1	27.2	67.3	65.7	79.3
**+1 ng IFN-g**	1	631,849	636,441	596,433	3291	5212	5805
	2	371,853	597,143	641,127	6061	8127	7446
	3	384,973	527,285	498,277	5927	15,056	19,497
	4	409,159	461,234	451,065	12,310	19,702	26,071
Mean ± SEM		449,459 ± 61,286	555,526 ± 38,695	546,726 ± 43,666	6897 ± 1914	12,024 ± 3288	14,705 ± 4865
CV		27.3	13.9	16.0	55.5	54.7	66.2
**+10 ng IFN-g**	1	757,165	763,603	748,453	3381	5834	8353
	2	390,485	640,182	606,920	5970	8136	7781
	3	412,008	563,646	539,970	5932	13,927	18,633
	4	394,570	462,345	414,055	13,533	19,158	23,188
Mean ± SEM		488,557 ± 89,658	607,444 ± 63,528	577,350 ± 69,650	7204 ± 2195	11,764 ± 2996	14,489 ± 3824
CV		36.7	20.9	24.1	60.9	50.9	52.8

^#^ Absolute MFI values for W6/32 and HC-10 in non-dividing CD3+ T cells (NDCs), total dividing CD3+ T cells (DCs) and most-dividing CD3+ cells (MDCs) are indicated as arbitrary units of fluorescence (auf). SEM, Standard Error of the Mean. CV, coefficient of variation. All amounts of cytokines indicated are in ng/mL. Statistically significant (*p* < 0.05) values between DC and MDC in relation to NDC are indicated by an asterisk.

## Data Availability

The original contributions presented in the study are included in the article. Further inquiries can be directed to the corresponding author.

## Supplementary Materials

The supporting information can be downloaded at: https://www.mdpi.com/article/10.3390/ijms25179376/s1.
