# Supplementary material for: IL-10 and TGF-β, but Not IL-17A or IFN-γ, Potentiate the IL-15-Induced Proliferation of Human T Cells: Association with a Decrease in the Expression of β2m-Free HLA Class I Molecules Induced by IL-15
Source: Int J Mol Sci. 2024 Aug 29;25(17):9376. doi: 10.3390/ijms25179376 (PMC11394758; doi:10.3390/ijms25179376)
Supplement: Supplementary file 1 [file ijms-25-09376-s001.zip › Suppl. Figures Legends.pdf]

**SUPPL. FIGURES LEGENDS**

**Suppl. Figure S1. Effect of IL-15 alone or in combination with IL-10, TGF-β, IL-17A or IFN-γ on the relative percentage of CD3+CD8+ and CD3+CD8− T cells.**
Fresh PBL were isolated and cultured as indicated in the legend of Figure 1 in the absence or presence of the four different cytokines. Twelve-day activated PBL were labelled with W6/32+GAM-PE or HC-10+GAM-PE, followed by anti-CD3 and anti-CD8β antibodies, as indicated in the Material and Methods, and analyzed by flow cytometry. (**A**) The graph shows the relative percentage of dividing CD3+CD8+ T cells and CD3+CD8− T cells (Mean±SEM, n=4) on gated lymphoblasts in the culture conditions indicated. (**B**) The graphs show the relative percentage of dividing CD3+CD8+ T cells (Mean±SEM, n=4) on gated lymphoblasts in the culture conditions indicated. P values are indicated.

**Suppl. Figure S2. Effect of IL-15, TGF-β, IL-17A and IFN-γ on the expression of W6/32 and HC-10 epitopes.**
Fresh PBL were isolated and cultured as indicated in the legend of Figure 1 in the absence or presence of IL-17A and IFN-γ. Twelve-day activated PBL were labelled with W6/32+GAM-PE or HC-10+GAM-PE, followed by anti-CD3 and anti-CD8β antibodies, as indicated in the Material and Methods, and analyzed by flow cytometry. (**A**) Graph shown the absolute HC-10 MFI values in non-dividing CD3+ T cells (NDC), total dividing CD3+ T cells (DC) and the most dividing CD3+ T cells (MDC) in four different experiments, after culture with IL-15. (**B**) Histogram of a representative experiment showing the emission fluorescence curves for W6/32, HC-10 and an irrelevant IgG2a antibody in IL-15-activated CD3+ T cells. (**C**) Graph shows the normalized MFI values for W6/32 (black bars) and HC-10 expression (grey bars) in the pool of the most dividing CD3+ T cells, CD3+CD8+ T cells and CD3+CD8− T cells. (**D**) Graphs show the normalized HC-10 MFI values in CD3+ T cells, CD3+CD8+ T cells and CD3+CD8− T cells in cultures with IL-15+IL-17A (upper row) and with IL-15+IFN-γ (lower row). P values are indicated.
