# Supplementary figures and images for: IL-10 and TGF-β, but Not IL-17A or IFN-γ, Potentiate the IL-15-Induced Proliferation of Human T Cells: Association with a Decrease in the Expression of β2m-Free HLA Class I Molecules Induced by IL-15
Source: Int J Mol Sci. 2024 Aug 29;25(17):9376. doi: 10.3390/ijms25179376 (PMC11394758; doi:10.3390/ijms25179376)

## Slide 1
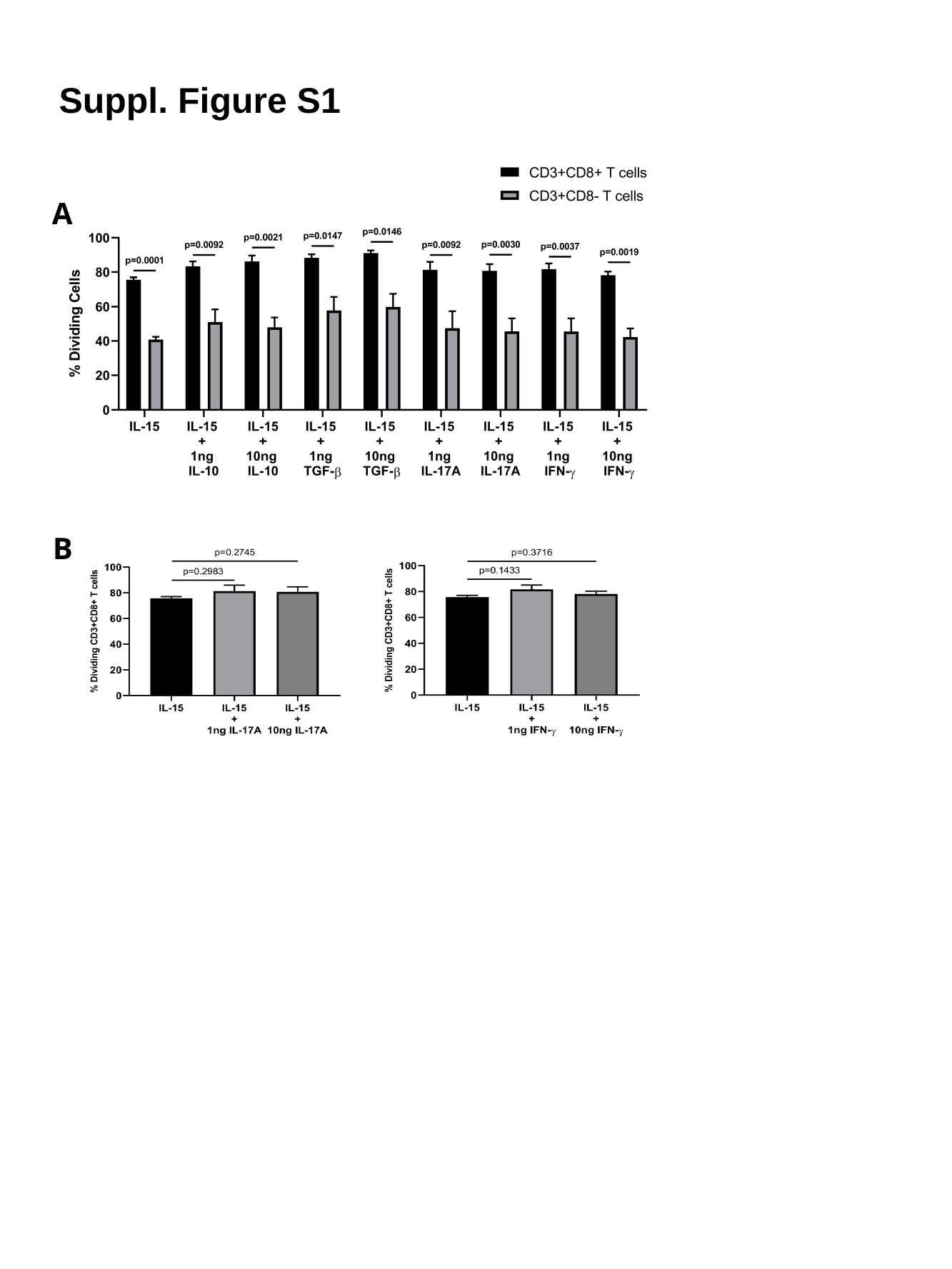

Suppl. Figure S1
A
B

Supplement: Supplementary file 1 [file ijms-25-09376-s001.zip › Suppl. Figure S1.pptx]

## Slide 1
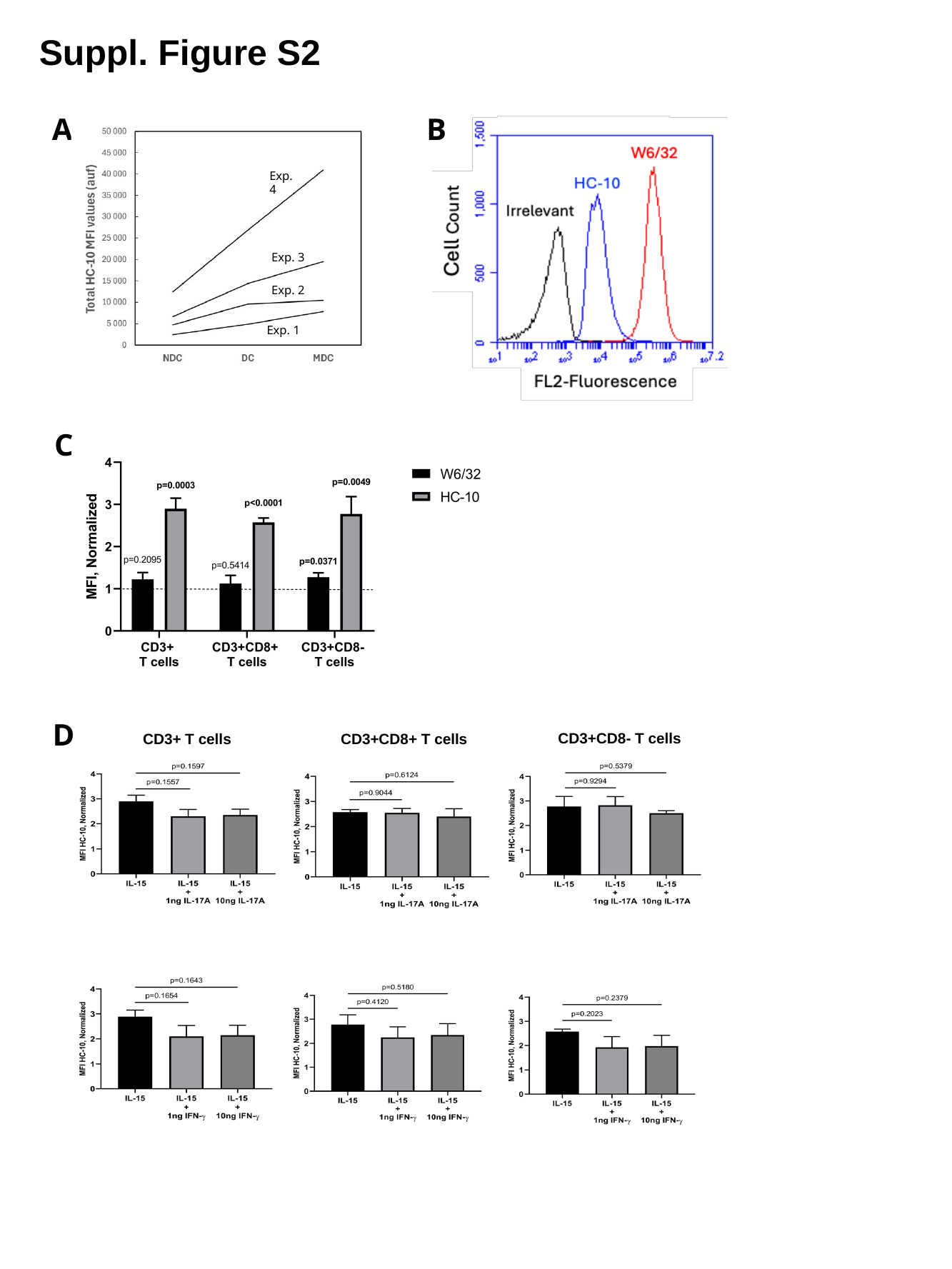

Suppl. Figure S2
A
B
Exp. 4
Exp. 3
Exp. 2
Exp. 1
C
D
CD3+CD8- T cells
CD3+ T cells
CD3+CD8+ T cells

Supplement: Supplementary file 1 [file ijms-25-09376-s001.zip › Suppl. Figure S2.pptx]
